# Inadequate Personal Protective Equipment Factors and Odds Related to Acute Pesticide Poisoning: A Meta-Analysis Report

**DOI:** 10.3390/ijerph21030257

**Published:** 2024-02-23

**Authors:** Dorothy Nguyen, Candace S. J. Tsai

**Affiliations:** Department of Environmental Health Sciences, Fielding School of Public Health, University of California, Los Angeles, Los Angeles, CA 90095, USA; dorothynguyen@ucla.edu

**Keywords:** PPE, pesticide poisoning, acute exposure, odds ratio

## Abstract

Introduction: Acute pesticide poisoning (APP) continues to affect farm workers, especially in low- and middle-income countries (LMIC). The dose–response relationship between exposure and APP is well-researched, but pesticide exposure assessment in a practical environment is difficult to perform, considering various work practices and protections in place. It is well known that inadequate personal protective equipment (PPE) use is a risk factor of APP. However, it is unknown which types of inadequate PPE use, such as face or other types of general protection, are most harmful. Methods: This study aimed to identify if inadequate PPE use is an indicator of APP risk following established specifications for meta-analysis of epidemiological studies. Included studies reported an odds ratio (OR) between PPE use to APP in agricultural workers. Data extracted from selected articles included authors, publication year, country of origin, farm type, population size, method of data collection and time frame of reported symptoms, job task, type of PPE and pesticides used, adjustments made in analysis, OR for APP, and 95% confidence intervals (CI). Meta-analysis was performed using a random effects model, where ORs were pooled to assess an overall estimate for poisoning odds. Results: Our findings suggested that inadequate PPE use was associated with increased odds (OR = 1.57, 95% CI = 1.16–2.12) of having APP. Failure to use general protection and inadequate face protection increased odds of APP by 1.29 times (95% CI = 0.88–1.90) and 1.92 times (95% CI = 1.23–3.00), respectively. Conclusions: The meta-analysis results indicate that improper facial protection and general protection are not differently associated with APP odds. Our study concludes that more robust protection against inhalation and dermal contact are critical because any gaps in comprehensive full-body PPE would put workers and exposed populations at APP risk.

## 1. Introduction

Chemical pesticides have drastically increased agricultural output to support growing populations worldwide by increasing land productivity, increasing food quality, and reducing crop losses. Global agricultural use of pesticides in 2020 was 2.7 million tons, which is a 60% increase from the 1.7 million tons in 1990 [1]. Pesticide usage has plateaued since 2010, and is predicted to stabilize or decrease because of efforts to reduce usage in China, the European Union, and elsewhere [1]. However, decreased pesticide usage is not anticipated in all countries. In particular, some estimates project that pesticide usage will increase in low-income countries [2].

Chemical pesticides have health consequences for exposed workers and communities. Many chemical pesticides are known to cause neurological, respiratory, cardiovascular and muscular symptoms, depending on the specific pesticide or pesticide class. For example, organophosphates interrupt the cholinergic pathway responsible for muscle contraction. This can manifest in Parkinson’s disease, diarrhea, and muscle weakness. In contrast, pyrethroids bind to sodium channels to cause paresthesia, respiratory difficulty, and CNS damage [3]. Acute pesticide poisoning (APP) is an event where an illness or health effect is experienced within 48 h of exposure. Common symptoms include abdominal cramps, muscle weakness, cough and headache [4].

It is estimated that there are 1.8 billion agricultural workers [5] and 385 million annual cases of APP worldwide, 11,000 of which result in death [6]. APP occurs most commonly in low- and middle-income countries (LMIC) today [7,8,9]. It is estimated that 0.05% of workers in the US have experienced non-fatal acute pesticide poisoning, compared to 84% in Burkina Faso [7]. Non-fatal APP is estimated to be greatest in south Asia, followed by southeast Asia and east Africa [6].

High rates of APP in LMICs may continue to occur because of increased pesticide exposure from lack control strategies. Examples include pesticide-related injury reporting, government regulation of highly toxic chemicals, personal protective equipment (PPE), training, and understanding of pesticide labels [10]. Furthermore, LMICs have higher proportions of farmers that are small-scale, so they may live in closer proximity to pesticides and have less resources for controlling exposure [11,12].

Hats, face shields, goggles, and respirators are primary protection against facial contact and inhalation of chemicals. Body protection like coveralls, aprons, gloves, and footwear protect workers against exposure to skin, daily clothing, and transfer of chemicals to the face for ingestion or inhalation [13]. The primary route of exposure and uptake of pesticides is dermal absorption followed with inhalation [14,15,16]. The face is more sensitive to dermal uptake. The eyes, scalp, forehead, and ear canal have 3–5 times the absorption rates for parathion than the forearm [17,18]. Hence, the use of facial PPE that prevents dermal contact may be critical to the prevention of APP.

The association between pesticide exposure and disease is well-established, but knowledge about visible risk factors for APP, such as the use of specific pieces of PPE, may be more useful for worker training and education. This risk factor could be more practical in prevention of poisoning than assessing exposures directly. Quantitative exposure assessment is not a routine practice on farms, and many workers in LMICs do not know the exact chemicals they use. Studying symptomatic APP outcomes provides an easily identifiable endpoint compared to subclinical endpoints. For example, biomarkers are more invasive and expensive to identify.

While PPE use is a standard recommendation for workers exposed to pesticides, this research seeks to discern whether inadequate types of PPE is associated with APP. Face protective equipment and unspecified general PPE will be compared by reviewing and analyzing trends and odds reported by epidemiological studies that assess both PPE and symptoms of APP. Published studies with meta-analyses are limited and focus on exposure-based risk factors and chronic or subclinical health outcomes [19,20,21,22]. Existing epidemiological studies document several behavioral variables, but PPE types have not been compared. This meta-analysis bridges the gap between PPE behaviors and acute health outcomes in real work environments.

## 2. Methods

This study aimed to determine whether lack of PPE use is a risk factor for APP in the work environment. More specifically, this research explores whether inadequate face protection is associated with greater risk of APP than inadequate use of general PPE.

The review was not limited by publication year, in order to capture the entire history of chemical pesticide use in agricultural settings. This includes the rise of pesticide use as early as the early 20th century. Observational epidemiological studies were evaluated to identify associations between workers’ PPE practice and APP. The type of observational study including cross-sectional, cohort, and case–control studies was not limited. The population of interest was agricultural workers.

### 2.1. Selection Criteria

A literature search was conducted following established specifications for meta-analysis of observational studies in epidemiology (MOOSE) [23]. The electronic databases searched were PubMed and EMBASE. These databases were selected to provide adequate recall and coverage of studies [24]. The search terms were selected to reflect the outcome of interest, the work activities and study design type. The following keywords were searched: ((“acute pesticide poisoning” OR “pesticide poisoning” OR “pesticide health conditions”) AND (farmer OR occupational) AND (handling OR practice OR applicator OR PPE) AND (“odds ratio” OR “relative risk”)). The searches were performed during 12 November 2023–24 November 2023.

The following inclusion criteria was applied: (1) peer-reviewed English-language articles; (2) samples of agricultural workers; (3) reporting face protection equipment or unspecified general PPE use and APP symptoms defined as “any illness or health effect resulting from suspected or confirmed exposure to a pesticide” by the WHO [4]; and (4) estimated odds ratio (OR) or relative risk (RR) with 95% confidence interval (CI). Studies that were excluded had: (1) no OR or RR reported or insufficient data to calculate one; (2) non-symptomatic endpoints, including biomarkers like lung function metrics, blood, serum and urine metabolites, and ergonomic ratings, as these are not as readily identifiable as APP, so are beyond the scope of this review; and (3) reported behavior-based traits including knowledge, attitudes, and practices outcomes as those are not APP outcomes.

### 2.2. Data Extraction

Data extracted from the studies included: the authors, publication year, country of origin, farm type, sample size, job task, pesticides to which participants were exposed, method of data collection utilized by the researchers, time period of symptoms, type of PPE worn by participants, reported APP symptoms, adjustments for confounding factors made in statistical analysis, OR or RR for APP, and 95% confidence intervals (CI).

### 2.3. Statistical Analysis

Meta-analysis was performed using a random effects model on the statistical program STATA 17 to obtain pooled OR values, reflecting the odds of an APP symptom associated with the failure to use of PPE. The “face” protection subgroup included eye protection and masks use recorded in studies, and the “general” protection subgroup included studies that did not detail specific PPE articles. The weight of a study in a random effects model is inversely related to error variance [25]. Heterogeneity was assessed with the I^2^ statistic and publication bias was assessed with Bregg’s funnel. Difference between the subgroups was assessed with Cochran’s Q statistic.

## 3. Results

### 3.1. Screening

**Figure 1:**
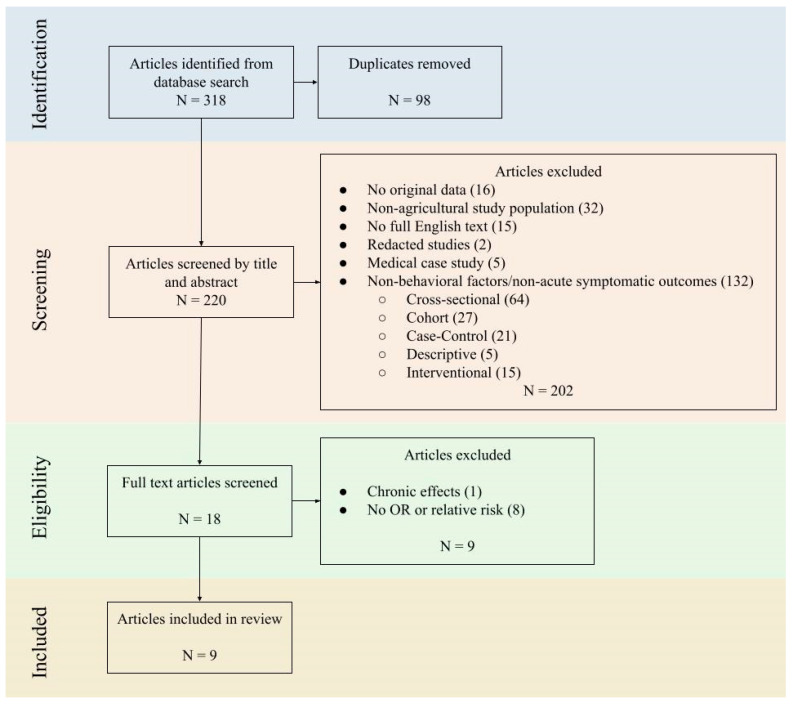
Flow chart of article selection.

Articles were identified from the literature search, and duplicates were removed (Figure 1). Articles from the search were dated as early as 1971, and included various study designs including cross-sectional, cohort, case–control, descriptive, and interventional studies. Articles were then screened by title and abstract before being excluded according to the inclusion and exclusion criteria. Although the search yielded articles as far back as the 1970s and multiple study types, many of these did not meet the subsequent criteria for exposure related to face or general PPE and outcome of APP. Article exclusion and selection is detailed in Figure 1.

### 3.2. Studies Included in Meta-Analysis

Nine studies were selected based on the pre-defined inclusion and exclusion criteria (Figure 1), all of which were based on cross-sectional research design and reported ORs. Countries represented by these studies were Uganda, Turkey, Argentina, Italy, South Korea, Chile, Jamaica, and Indonesia. In regard to specific pieces of PPE reported, five explicitly reported PPE for the face (face mask or eye protection), which were categorized as “face” protection. Four studies did not detail the type of specific PPE articles, which were categorized as “general” protection. These typically included unspecified use of hats, boots, long clothing, and eye protection. Worker PPE usage and reported symptoms were typically obtained with questionnaires and interviews. All studies asked what PPE workers typically use. The time period for reported symptoms is included in Table 1. Although some studies reported on the chemical class or exact chemical agent that workers utilized, many did not specify the pesticides used or provided only a general class. An overview of the selected studies is listed in Table 1.

### 3.3. Meta-Analysis

**Figure 2:**
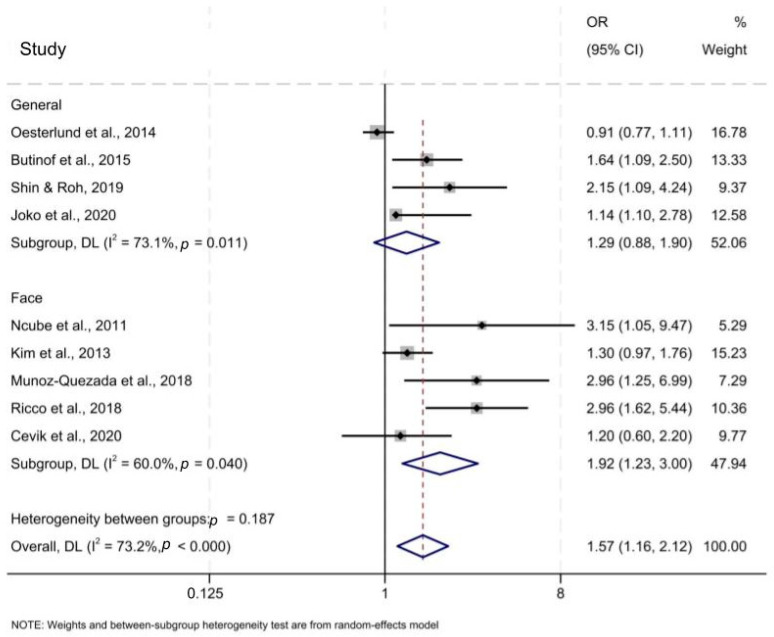
Summary of association between inadequate PPE and APP, separated by general and face protection categories [26,27,28,29,30,31,32,33,34].

Figure 2 demonstrates the association between inadequate face or general PPE and OR of having APP symptoms. The bold line at OR = 1 indicates the odds ratio at which inadequate and adequate PPE use have the same odds of APP. Left of the bold line where OR < 1 is where inadequate PPE is associated with lower odds of APP than adequate PPE. Right of the bold line where OR > 1 is where inadequate PPE is associated with higher odds of APP than adequate PPE. ORs reported by individual studies appear as lines and are analyzed into subgroups. Subgroups and overall effect appear as diamonds. The pooled OR of overall inadequate PPE, encompassing both the general and face protection subgroups, was 1.57 (95% CI 1.16–2.12) compared to the reference group of workers using adequate or complete PPE. This suggests that inadequate PPE use is associated with increased odds of APP. There is slight variation in OR depending on PPE type. Inadequate general protection had OR = 1.29 (95% CI 0.88–1.90) and inadequate facial protection had OR = 1.92 (95% CI 1.39–1.89). These ORs suggest that not wearing face protection could be associated with higher odds of APP than not wearing general protection, although Cochran’s Q test indicated that the difference is not statistically significant.

**Figure 3:**
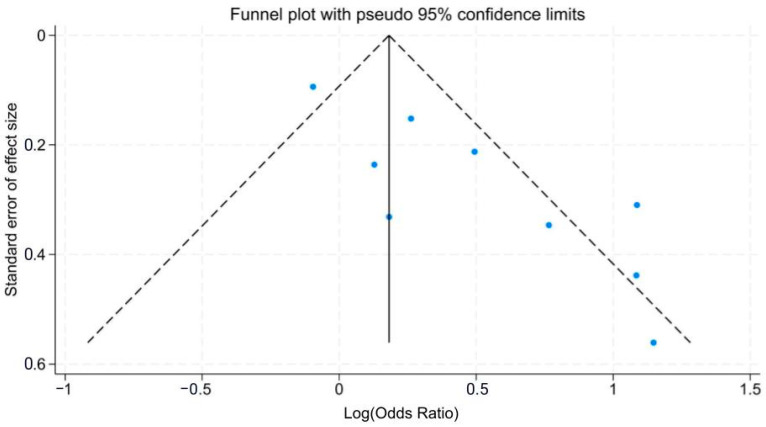
Begg’s Funnel plot of studies included in the meta-analysis of PPE and the odds of APP [26,27,28,29,30,31,32,33,34]. Each study’s OR and standard error is represented by a blue point.

Begg’s asymmetry method indicated publication bias (Figure 3). Each study is represented by a point. Symmetrical distribution of the points around the center line of the funnel indicates unbiased publication of both significant (right of the funnel center) and negative findings (left of the funnel center). There are six studies on the positive side of the funnel, two on the negative side, and one in the center. The asymmetrical distribution suggests publication bias for studies with significant findings.

## 4. Discussion

### 4.1. Predominant Routes of Exposure

Dermal contact is the primary route of pesticide exposure [14,15,16], so this study is focused on PPE that provides dermal exposure protection. However, adherence to recommended PPE for all exposure routes is needed for robust protection, because pesticide exposure can also occur through ingestion and inhalation. The varying degrees of protection against APP provided by PPE worn on different body parts may reflect differences of magnitude of dermal exposure across the body and the effectiveness of the specific pieces of PPE worn [17]. The present meta-analysis found that inadequate face protection may have an inclination for higher APP risk than inadequate general protection.

Dermal exposure to pesticides depends on pesticide deposition on the body, the rate of pesticide uptake, and the protective ability of PPE. The magnitude and location of pesticide deposition depends on work characteristics, such as crop and sprayer type. The extremities are typically most contaminated [35]. Studies in greenhouses show that potential exposure is higher to lower limbs (feet and legs) [36], but lower on the arms, chest, and back [36,37,38]. Paddy workers and vegetable sprayers also experience high pesticide exposure to the lower extremities during spraying [39,40,41]. High deposition to the lower limbs suggests that aprons, pants, and proper footwear are important PPE to reduce APP risk. Pesticide uptake through the skin varies across the body [42], especially at the face. Portions of the head and face, for example, have relative absorption rates that are 3.7 to 5.4 times that of the forearm for parathion, respectively [17,18]. Eyes have the most elevated sensitivity and absorption ability [13]. This could indicate that eye protection may be more critical than facial or body coverings against dermal exposure alone.

Acute and chronic toxicity of inhaled pesticides can occur [43,44,45] despite inhalation risk being lower in open-field farms with traditional spraying methods that do not generate small pesticide droplets [14]. The lethal dose, 50% (LD50), the dose at which 50% of a test population dies [46], of acute toxicity of pesticides for inhalation is lower than both the oral and dermal LD50s. Masks, as reported by one study included in this meta-analysis [32] and similar epidemiological studies in LMIC [31,47,48], could prevent dermal face exposure, but do not protect the respiratory tract like wearing a respirator. PPE protecting against dermal exposure is a priority in reducing APP risk, but lack of inhalation protection further worsens APP.

The burden of APP among workers exposed to pesticides in LMICs is compounded by the growing use of pesticides, lack of training, and inaccessibility to chemical-specific PPE recommendations that target all potential exposure routes [11,12]. The overall OR = 1.57 (95% CI: 1.16–2.12) indicates that failure to wear PPE in general is associated with increased APP risk. All studies note that many workers experience APP. Partial PPE measures protecting only some parts of the body may still be associated with APP because of the chemical potency of pesticides and multiple possible exposure routes. The similar face and general subgroup ORs may suggest that “partial protection measures” may not be associated with complete protection against exposure and health outcomes, further highlighting that comprehensive PPE is essential.

### 4.2. Challenges to Pesticide Safety

Lack of existing and accessible knowledge of pesticide handling and health effects contribute to APP in LMICs. While farmers are aware that pesticides can be dangerous, many are not aware of specific chemicals used, manufacturers’ instructions [49], routes of exposure [50], and safe storage and disposal practices [51]. Higher levels of education and literacy are well-documented to have a negative association with poisoning and positive association with knowledge and practices [31,52,53,54,55]. Education continues to be a hurdle, especially for small scale farmers. Furthermore, there are cost restraints and comfort concerns [56] that make it difficult to adhere to robust PPE.

Currently, exposure measurements and biomarkers are used to assess the risk of ill health, including APP [57,58]. However, quantifying exposure requires identification of the chemical mixtures used, sampling equipment, and costly analysis that are not feasible in many LMICs or smaller farms. Biomarker monitoring involves repeated follow-up, farmer buy-in and is potentially invasive. As such, the behavioral association between PPE use and APP found in the present study could inform PPE types to prioritize. Inadequate face protection in the present study was associated with double the odds of having APP compared to adequate face protection. Lack of eye protection and face masks is more risky than inadequate general PPE.

### 4.3. Limitations

The epidemiological studies assessed in this meta-analysis have variability in PPE and pesticide risk factors (Table 1). Specific types of PPE considered in OR calculations differ. Some studies specify eye or face protection [30,31,32,33,34], while others only report if PPE is used or not [26,27,28,29]. Pesticides that workers use are reported as specific chemicals [30,32], target organisms (herbicide, insecticide, fungicide) [27,29], the WHO toxicity class [26], or not at all [28,31,33,34]. ORs are calculated based on the presence of absence of any APP symptoms, rather than specific symptoms or symptoms affecting human organ systems. In brief, risk factors and outcomes are reported nonuniformly or vaguely between studies.

These limit more granular findings that could be more chemical-specific or human organ system-specific, such as being able to associate if certain inadequate PPE types are related to symptoms that are dermal, gastrointestinal, neurological, or musculoskeletal. Many studies do not report the same chemical classes or hazard classifications, and some do not report any chemicals because workers are unfamiliar with what pesticides are used. As such, the present study’s pooled ORs only reflect unspecified or general pesticides. However, chemicals have specific PPE recommendations [59,60]. Certain common chemicals, like organophosphates and carbamates, have profound health effects through dermal uptake, while glyphosate and pyrethroids can cause more serious symptoms from inhalation [4]. The prevalent practice of mixing multiple pesticides and workers’ unfamiliarity with the chemicals used further stresses the importance of comprehensive PPE. Eye and mask protection need to be prioritized due to the face’s dermal, inhalation, and ingestion vulnerability.

All the literature identified in this review was cross-sectional studies, which cannot assess causation. Cross-sectional studies may be conducted more frequently because they are convenient, examine many determinants at once, and do not require follow-up with participants, which can be particularly challenging in LMICs. Other systematic reviews on chronic pesticide-related health outcomes, namely cancers, have not been able to cement conclusions [21], and required data was only found in cross-sectional studies [22]. Nonetheless, the approach in the present meta-analysis could be used to further parse out more specific associations to inform pesticide practices. If more studies detailing specific PPE practices become available, more powerful future association analysis may discern if certain PPE pieces like cloth masks and goggles are truly more protective than items like gloves, coveralls, and boots. The present study’s inadequate face protection group has a higher OR for APP compared to inadequate general protection, although the difference was not statistically significant. Future studies may be able to see a more profound difference between face protection and general protection and confirm the hint of direction found in this study.

Begg’s funnel plot (Figure 3) indicates that the studies are scattered asymmetrically, with six studies having an overall effect that is more positive and only two studies having an overall effect that is more negative. This may suggest that studies showing that inadequate PPE is more strongly associated with APP are more likely to be published than studies that do not have significant or negative findings [61]. The higher OR for the face group demonstrates the importance of mask and eye protective measures to worker health. The similarity between the general and face subgroups indicates that comprehensive full-body PPE is essential because pesticides are potent and have multiple routes of exposure. Lacking face protection like masks, eye or head protection may put users at increased odds of APP because of susceptibility to various routes of exposure are heightened around the face and respiratory area, including dermal contact and inhalation [13]. Meanwhile, body protection like gloves and clothing can protect parts of the body that experience high pesticide dermal deposition and reduce transfer to the vulnerable face areas. 

## 5. Conclusions

Workers in LMICs are still at high risk of APP. Abundant research has demonstrated that pesticide exposures can lead to acute and chronic health effects, but the direct assessment of exposures can be difficult on farms in LMICs compared to using easily identifiable risk factors such as PPE. The meta-analysis results in this study suggest that the practice of PPE is beneficial, as inadequate use of PPE puts users at higher the odds of APP (OR = 1.57, 95% CI = 1.16–2.12). Inadequate face protection of eye protection and masks has a tendency for higher APP odds (OR = 1.92, 95% CI = 1.23–3.00) than inadequate general protection (OR = 1.29, 95% CI = 0.88–1.90). Proper face protection can more likely reduce potential health effects because areas of the face experience accelerated dermal update of pesticides. Pesticide strength and entry through multiple pathways necessitate robust and comprehensive PPE measures. These findings could inform decisions on changes in operating practices that are reasonable with worker attitudes and resources.

## Figures and Tables

**Table 1:** Summary of selected studies by broad PPE type.

Study	Sample Information	Pesticides Reported	Method	Time Period of Symptoms	OR Adjustments
General					
Osterlund et al., 2014 [26]	Uganda—small-scale vegetable farms; 317 farmers; APP case count not specified	WHO Classes I, II, III, U—mostly Class II	Interview and questionnaire assessing knowledge, attitudes, symptoms, hygiene, and PPE defined as wearing one or more vs. having no precautions of gloves, overalls, boots, mask, hat, long-sleeved shirt *	Symptoms experienced “immediately” after spraying in the last year	District, age gender, marital status farmer group, education level, use of PPE and precaution
Butinof et al., 2015 [27]	Argentina—extensive crop farms; 880 applicators; 417 reported APP	Various chemical herbicides, insecticides, and fungicides	Self-administered questionnaire assessing sociodemographic factors, work practices, and PPE defined as “adequately protected” with at least 90% PPE vs. “without proper protection” *	Symptoms that appear “after the beginning of the exposure”	Irritation symptoms, medical consultation and hospitalization
Shin and Roh, 2019 [28]	South Korea—orchards; 394 farmers; 323 reported APP	Not specified	Questionnaire and interview assessing sociodemographic factors, farming occupational exposure factors including APP, disease history, lifestyle factors, and PPE defined as 4–7 pieces of PPE worn vs. 0–3 pieces including goggles, hat, boots, gloves, mask, clothing [top], and clothing [bottom] *	Symptoms within 48 h of exposure within the last year	Gender, age, educational status, smoking, and drinking status
Joko et al., 2020 [29]	Indonesia—red onions, 100 farmers; 17 reported APP	Mixtures of insecticides and fungicides	Self-administered questionnaire assessing acute symptoms and PPE usage defined as “Using PPE” wearing any number of boots, gloves, glasses, trousers, and long-sleeved clothes vs. “Using no PPE” *	Not specified	None
Face					
Ncube et al., 2011 [30]	Jamaica—yam, banana, dasheen, cane tomato, pepper, plantain, and corn; 359 farmers; 57 reported APP	Herbicides (paraquat, 2,4D, ametryin, ioxynil, terbutryn); fungicides (glyphosphate, copper hydroxide); insecticides (cyhalothrin, deltamethrin, diazinon)	Interviewer-administered questionnaire assessing knowledge and practice regarding handling, use, storage and disposal of pesticides, self-reported acute symptoms, and PPE usage defined as “Always” using a mask/respirator when handling pesticides vs. “never” ‡	Acute symptoms experienced within the last 2 years	Not specified
J.-H. Kim et al., 2013 [31]	South Korea—rice, vegetable, greenhouse, fruit, mixed and other; 1958 male farmers; 449 reported APP	Not specified	Survey and interviews assessing poisoning, type of treatment, and PPE defined as use of safety glasses or no use ‡	Symptoms experience within 48 h of pesticide use	Age, income
Muñoz-Quezada et al., 2017 [32]	Chile—114 agricultural workers, 93 non-agricultural workers; APP case count not specified	Organophosphates, herbicides, pyrethroids, fungicides, other, unspecified	Questionnaire assessing exposure history, symptoms, and PPE defined as use of respiratory PPE or no use †	“Symptoms associated with recent poisoning by exposure to pesticides”	None
Riccó et al., 2018 [33]	Italy—open and closed-field fruits, vegetables, and flower farms; 260 applicators; 113 reported APP	Not specified	Questionnaire assessing knowledge, attitudes, practices, health, and PPE defined as use of eye mask or no use ‡	Frequency of experiencing symptoms when handling pesticides	Age, sex and ethnicity
Cevik et al., 2020 [34]	Turkey—fruit and vegetable farms; 565 applicators; 64 reported APP	Not specified	Structured questionnaire assessing sociodemographic factors, preventative measures, and PPE defined as use of eye mask or no use ‡	“Two or more symptoms that occur within 48 h of spraying in the past year”	None specified

* Specific PPE was not specified (general), † Specific PPE reported were face masks, ‡ Specific PPE reported was eye protection.

## Data Availability

The data information and STATA code can be requested through corresponding author.
